# The impact of elective surgery postponement during COVID-19 on emergency bellwether procedures in a large tertiary centre in Singapore

**DOI:** 10.1093/intqhc/mzae022

**Published:** 2024-03-20

**Authors:** Sze Ling Chan, Alwin Yaoxian Zhang, Sean Shao Wei Lam, Vijaya Rao, Devendra Kanagalingam, Hiang Khoon Tan, Pierce Kah Hoe Chow, Sachin Mathur

**Affiliations:** Health Services Research Centre, SingHealth, 20 College Road, Academia, Level 6, Singapore 169856, Singapore; Health Services & Systems Research, Duke–NUS Medical School, 8 College Road, Singapore 169857, Singapore; Division of Surgery & Surgical Oncology, National Cancer Centre Singapore and Singapore General Hospital, 30 Hospital Boulevard, Singapore 168583, Singapore; Health Services Research Centre, SingHealth, 20 College Road, Academia, Level 6, Singapore 169856, Singapore; Health Services & Systems Research, Duke–NUS Medical School, 8 College Road, Singapore 169857, Singapore; SingHealth Duke–NUS Global Health Institute, 8 College Road, Singapore 169857, Singapore; International Collaboration Office, SingHealth, 168 Jalan Bukit Merah, #11-01 Surbana One, Singapore 150168, Singapore; Division of Surgery & Surgical Oncology, National Cancer Centre Singapore and Singapore General Hospital, 30 Hospital Boulevard, Singapore 168583, Singapore; Department of Obstetrics & Gynaecology, Singapore General Hospital, 20 College Road, Academia, Level 5, Singapore 169856, Singapore; Division of Surgery & Surgical Oncology, National Cancer Centre Singapore and Singapore General Hospital, 30 Hospital Boulevard, Singapore 168583, Singapore; SingHealth Duke–NUS Global Health Institute, 8 College Road, Singapore 169857, Singapore; Duke Global Health Institute, 310 Trent Drive, Durham, NC 27710, USA; Division of Surgery & Surgical Oncology, National Cancer Centre Singapore and Singapore General Hospital, 30 Hospital Boulevard, Singapore 168583, Singapore; Surgery Academic Clinical Program, Duke-NUS Medical School, 8 College Road, Singapore 169857, Singapore; Division of Surgery & Surgical Oncology, National Cancer Centre Singapore and Singapore General Hospital, 30 Hospital Boulevard, Singapore 168583, Singapore; Department of General Surgery, Singapore General Hospital, 20 College Road, Academia, Level 5, Singapore 169856, Singapore

**Keywords:** acute care surgery, pandemic preparedness, health services research, health resources

## Abstract

The coronavirus disease 2019 (COVID-19) pandemic drove many healthcare systems worldwide to postpone elective surgery to increase healthcare capacity, manpower, and reduce infection risk to staff. The aim of this study was to assess the impact of an elective surgery postponement policy in response to the COVID-19 pandemic on surgical volumes and patient outcomes for three emergency bellwether procedures. A retrospective cohort study of patients who underwent any of the three emergency procedures [Caesarean section (CS), emergency laparotomy (EL), and open fracture (OF) fixation] between 1 January 2018 and 31 December 2021 was conducted using clinical and surgical data from electronic medical records. The volumes and outcomes of each surgery were compared across four time periods: pre-COVID (January 2018–January 2020), elective postponement (February–May 2020), recovery (June–November 2020), and postrecovery (December 2020–December 2021) using Kruskal–Wallis test and segmented negative binomial regression. There was a total of 3886, 1396, and 299 EL, CS, and OF, respectively. There was no change in weekly volumes of CS and OF fixations across the four time periods. However, the volume of EL increased by 47% [95% confidence interval: 26–71%, *P* = 9.13 × 10^–7^) and 52% (95% confidence interval: 25–85%, *P* = 3.80 × 10^–5^) in the recovery and postrecovery period, respectively. Outcomes did not worsen throughout the four time periods for all three procedures and some actually improved for EL from elective postponement onwards. Elective surgery postponement in the early COVID-19 pandemic did not affect volumes of emergency CS and OF fixations but led to an increase in volume for EL after the postponement without any worsening of outcomes.

## Introduction

The coronavirus disease 2019 (COVID-19) pandemic placed significant demands upon healthcare resources worldwide. Significant re-organizational efforts were necessary to increase intensive care unit (ICU) beds and manpower resources to treat COVID-19 patients [1]. Furthermore, imaginative protocols were required to triage patients, procure, and provide personal protective equipment to staff while maintaining reasonable inpatient and outpatient patient care [1–3]. Cancelling or postponing elective surgery (ES) was the most common measure taken, a move recommended by the US Centers for Disease Control and Prevention and several surgical societies in the early phase of the pandemic [4, 5].

Cancellation or postponement of ES is a delicate balance between beneficence and non-maleficence, while considering patient autonomy and justice [6]. Clinical urgency and need for postoperative intensive care support are key considerations when prioritizing patients for surgery [6, 7]. Orthopaedic surgeries are among the most cancelled or postponed, as many have low urgency [6, 8]. A systematic review found that along with the expected decrease in elective orthopaedic surgeries, the number of emergency and trauma surgeries also decreased, possibly due to a decrease in traffic and sports accidents during periods of lockdown [9]. The consequences for other postponed procedures may be more uncertain and variable. For example, with postponement of hernia surgeries, some centers saw an increase [10, 11], no change [12, 13] and others saw a decrease in emergency cases [14–16].

ES postponement, though mandated by the devastating resource constraint imposed by the pandemic, are likely to lead to collateral damage for patients’ health, function, and quality of life [5]. The outcomes would vary by type of procedure, the patient’s medical condition, and duration of delay. For example, patients awaiting total joint arthroplasty experienced increased joint pain and decreased function during the postponement of their procedures [17]. The emotional and psychological distress cannot be underestimated for patients caught between their physical suffering and the inpatient risk of contracting COVID-19, which drives them to delay rescheduling their surgery [17–19] . Other conditions can present in a more advanced stage, requiring more drastic treatments or leading to more complications. For example, a case series of emergency hernia procedures in the UK saw more need for intestinal resections and increased length of stay [10].

Lastly, healthcare systems face a significant burden in clearing the backlogs of postponed surgeries. An estimated 28 million ES were predicted to be cancelled or postponed worldwide during the peak of the COVID-19 pandemic, and these would take 45 weeks to clear based on a 20% increase in normal surgical volumes after the pandemic [20]. Now as we approach 4 years post-pandemic, there is a need to assess the longer-term outcomes of this approach to provide clear evidence to guide future resource planning strategies.

In Singapore, ES reduction was also adopted particularly during the ‘circuit-breaker’ or lockdown period [21]. In Singapore General Hospital (SGH), we developed an enhanced acute care surgery (eACS) service that comprised of five independent teams performing only emergency and trauma surgery separate from ES teams. New workflows were developed for patient movements from the ED, operating rooms, endoscopy, and radiology [22]. In the first 2 months of implementing the enhanced acute care surgery model, surgical volumes, length of stay (LOS), ED LOS, and time from surgery to discharge were significantly reduced compared to the corresponding 2 months in 2019, with no cross-infection of COVID-19 between staff and patients [22]. Research on the outcomes of ES postponement in Asia is sparse. The effects of ES postponement are also context-dependent and affected by an institution’s demographic, health system, and evolving COVID-19 policies. Singapore has implemented swift actions to contain the pandemic on all levels and achieved a very low COVID-19 mortality rate [23]. It is imperative to determine the consequences of some of these actions on non-COVID-19 outcomes to provide guidance for future pandemics for Singapore and health systems similar to ours.

The Lancet Commission on Global Surgery working group identified Caesarean section (CS), emergency laparotomy (EL), and open fracture (OF) fixation as the three bellwether procedures that most hospitals should be able to perform 24/7 and can be used as indicators of the adequacy of systems, resources, and skills needed to treat a broader range of essential surgical conditions [24].

The aim of this study was to assess the impact of an ES postponement policy in response to the COVID-19 pandemic on surgical volumes and patient outcomes for the three emergency bellwether procedures in the largest tertiary hospital in Singapore (SGH).

## Methods

### Setting and patient population

SingHealth is an academic medical centre and the largest of the three public healthcare clusters in Singapore. SGH is the largest tertiary hospital in SingHealth and in Singapore, with 1700 beds. This is a retrospective cohort study of patients who underwent any of the three emergency bellwether procedures (CS, EL, and OF fixation) in SGH between 1 January 2018 and 31 December 2021. There were no specific exclusion criteria. The list of included surgical procedures is listed in Supplementary Table S1. This study was reviewed and approved by the Centralized Institutional Review Board (CIRB Ref: 2021/2221). Waiver of informed consent was requested and approved as all data were de-identified. This study is reported according to the REporting of studies Conducted using Observational Routinely-collected Data (RECORD) statement (Supplementary File 1).

### Data source

The SingHealth Electronic Health Intelligence System (eHIntS) is an enterprise data repository that integrates information from electronic medical records and other healthcare transactional systems [25]. Access to eHIntS data for this research study was granted through institutional approval processes. Data on demographics, visit details, operation scheduling, peri-operative details, diagnosis, and discharge disposition were extracted from eHIntS for the included patients. Different data types were extracted from different subject areas within eHIntS and then linked via patient identifier and case number.

Records of procedures with missing priority (0.04%) were removed. Procedures that should be performed within 24 h (classified as ‘critical’, ‘early’, ‘immediate’, ‘emergency’, ‘semi-urgent’, ‘obstetric P1’, ‘obstetric P2’, ‘obstetric P3’, and ‘obstetric P4’) were classified as emergency procedures. Age was calculated using dates of birth and procedure, and race was recategorized into the four major groups in Singapore (“Chinese”, “Malay”, Indian’, and ‘Others’).

### Exposure and outcomes

**Figure 1:**
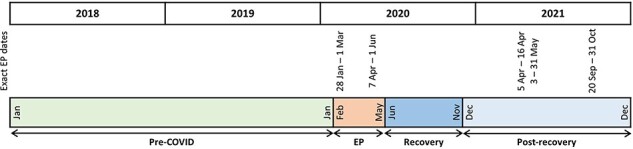
Timeline of elective surgery postponement and definition of time periods.

In SGH, reduction of ES began on 7 February 2020 and further reduction was mandated from 7 April 2020, which coincided with the beginning of the nationwide ‘circuit breaker’ period in Singapore [26, 27]. After the ‘circuit breaker’ period ended on 1 June 2020, ES was resumed (Fig. 1). Based on the simulation modelling results, operating room utilization was expected to increase immediately upon resumption from clearing the backlog and approach baseline by about 6 months after resumption of services [26].

We therefore divided the time periods into (i) pre-COVID (Jan. 2018–Jan. 2020), (ii) elective postponement (EP) (Feb.–May 2020), (iii) recovery (Jun.–Nov. 2020), and (iv) postrecovery (Dec. 2020–Dec. 2021) (Fig. 1). The outcomes include weekly surgical volume, LOS, ICU admission, ICU LOS, 30-day unplanned readmission, and 30-day mortality.

### Statistical analysis

All analyses were performed by type of procedure. A descriptive analysis of the time trend of surgical volumes and comparison of the distributions of weekly volumes across the four time periods using the Kruskal–Wallis test was first performed. Baseline characteristics of patients undergoing the three bellwether procedures in the four time periods were compared using the chi-squared test, one-way ANOVA, or Kruskal–Wallis test as appropriate. The effect of COVID time periods on outcomes were analyzed using median regression (for LOS and ICU LOS) and logistic regression, adjusting for baseline characteristics.

To evaluate the effects of EP, recovery period and postrecovery periods on surgical volumes, segmented negative binomial regression models were fitted to evaluate the change in level of each of these three periods compared to the pre-COVID period, with week of year included to control for seasonality.

To further examine trends of surgical characteristics over the intervention periods, the weekly volume was compared by the American Society of Anesthesiologists (ASA) score, a system for classifying patient’s pre-operative physical status [28]. For laparotomies, the weekly volume was also further analysed by a few key categories of postoperative diagnosis. We adjusted nine independent comparisons per procedure; therefore, a *P*-value of .002 was considered to be statistically significant. All analyses were conducted in R Version 3.5.1 [29].

## Results

From 2018 to 2021, there were a total of 3886, 1396, and 299 EL, CS, and OF fixations, respectively, that were performed in SGH. The breakdowns of the numbers of procedures and baseline characteristics by COVID time period are shown in Table 1.

**Table 1.** Baseline characteristics of patients by procedures and time period.

Characteristic	Pre-Covid	EP	Recovery	Postrecovery	*P*-value
EL, *n*	1899	283	557	1147	
Age, mean (SD)	56.3 (18.9)	53.0 (20.4)	58.5 (18.0)	57.7 (18.8)	**1.83** **×** **10^–4^**
Male gender, *n* (%)	1053 (55.5)	152 (53.7)	308 (55.3)	621 (54.1)	.874
Ethnicity, *n* (%)					.329
Chinese	1366 (71.9)	201 (71.0)	416 (74.7)	801 (69.8)	
Malay	154 (8.1)	25 (8.8)	46 (8.3)	91 (7.9)	
Indian	138 (7.3)	22 (7.8)	45 (8.1)	103 (9.0)	
Others	241 (12.7)	35 (12.4)	50 (9.0)	152 (13.3)	
ASA status, *n* (%)					**<2.2** **×** **10^–16^**
1	412 (21.7)	64 (22.6)	105 (18.9)	200 (17.4)	
2	1019 (53.7)	138 (48.8)	254 (45.6)	471 (41.1)	
3	369 (19.4)	64 (22.6)	142 (25.5)	305 (26.6)	
4	96 (5.1)	16 (5.7)	52 (9.3)	169 (14.7)	
5	3 (0.2)	1 (0.4)	4 (0.7)	2 (0.2)	
Emergency CS, *n*	770	111	188	327	
Age, mean (SD)	32.4 (5.0)	33.0 (4.4)	32.4 (4.8)	32.1 (4.6)	.388
Ethnicity, *n* (%)					.254
Chinese	376 (48.8)	55 (49.5)	77 (41.0)	138 (42.2)	
Malay	210 (27.3)	30 (27.0)	50 (26.6)	88 (26.9)	
Indian	91 (11.8)	12 (10.8)	31 (16.5)	53 (16.2)	
Others	93 (12.1)	14 (12.6)	30 (16.0)	48 (14.7)	
ASA status, *n* (%)					**<2.2** **×** **10^–16^**
1	572 (74.3)	37 (33.3)	59 (31.4)	84 (25.7)	
2	180 (23.4)	67 (60.4)	115 (61.2)	189 (57.8)	
3	14 (1.8)	7 (6.3)	11 (5.9)	28 (8.6)	
4	4 (0.5)	0	3 (1.6)	26 (8.0)	
Emergency of fixation, n	170	20	39	70	
Age, mean (SD)	45.2 (19.4)	39.0 (15.9)	47.2 (20.3)	42.8 (16.0)	.334
Male gender, *n* (%)	132 (77.6)	17 (85.0)	28 (71.8)	58 (82.9)	.492
Ethnicity, *n* (%)					.768
Chinese	78 (45.9)	13 (65.0)	22 (56.4)	33 (47.1)	
Malay	28 (16.5)	1 (5.0)	6 (15.4)	8 (11.4)	
Indian	33 (19.4)	3 (15.0)	5 (12.8)	14 (20.0)	
Others	31 (18.2)	3 (15.0)	6 (15.4)	15 (21.4)	
ASA status, *n* (%)					.004
1	71 (41.8)	15 (75.0)	17 (43.6)	38 (54.3)	
2	94 (55.3)	3 (15.0)	18 (46.2)	24 (34.3)	
3	5 (2.9)	1 (5.0)	3 (7.7)	6 (8.6)	
4	0	1 (5.0)	1 (2.6)	2 (2.9)	

Pre-COVID (Jan. 2018–Jan. 2020), EP (Feb.–May 2020), Recovery (Jun.–Nov. 2020), Postrecovery (Dec. 2020–Dec. 2021). Bold indicates statistically significant associations.

Abbreviation: SD: standard deviation.

### Emergency laparotomy

Patients who underwent EL during EP were younger (mean age 53.1 versus 56.3–58.5 years in other periods) (Table 1). There was also an increase in the proportion of patients with ASA 3 from EP onwards (22.6–26.6% versus 19.4% pre-COVID), and more patients with ASA 4 in the recovery and postrecovery periods (9.3% and 14.7% versus 5.1% and 5.7% in the pre-COVID and EP periods, respectively) (Table 1, Supplementary Figure S1 and Supplementary Table S1).

**Figure 2:**
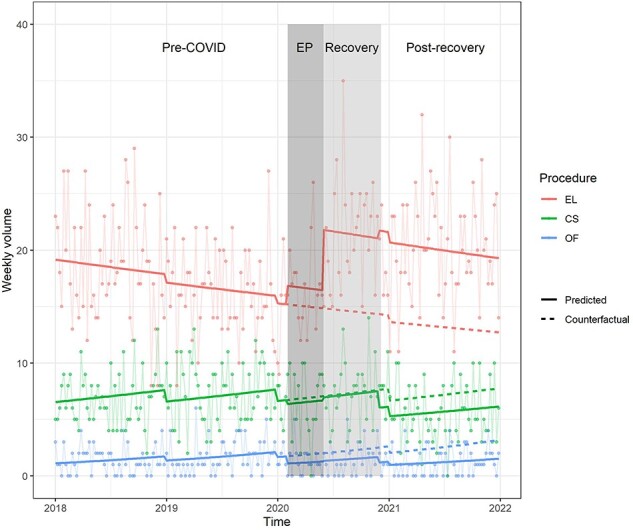
Actual and predicted volumes of the three bellwether procedures over time.

The weekly volume of EL remained fairly stable but increased by 47% [95% confidence interval (CI): 26–71%, *P* = 9.13 × 10^–7^) and 52% (95% CI: 25–85%, *P* = 3.80 × 10^–5^) in the recovery and postrecovery period, respectively (Fig. 2, Table 2).

The actual weekly volumes (points, joined by thin lines) are plotted over time by procedure. The thick solid lines represent the predicted volume from the segmented negative binomial regression models for each procedure and priority. The dotted lines represent the counterfactual predicted volumes if EP (and the resultant recovery and postrecovery periods) did not occur. Pre-COVID (Jan. 2018—Jan. 2020), EP (Feb.—May 2020), Recovery (Jun.—Nov. 2020), Post-recovery (Dec. 2020—Dec. 2021).

**Table 2.** Weekly volumes and interrupted time series regression results by procedure.

	Pre-COVID	EP		Recovery		Postrecovery		
Surgery	Median (range)	Median (range)	IRR (95% CI)	Median (range)	IRR (95% CI)	Median (range)	IRR (95% CI)	*P*-value^a^
EL	17 (8–29)	16 (12–26)	1.108 (0.936–1.310)	22 (15–35)	**1.468 (1.260–1.712)**	20 (11–32)	**1.517 (1.245–1.852)**	**2.01** **×** **10^–5^**
Emergency CS	7 (2–13)	8 (0–10)	0.948 (0.729–1.227)	7 (3–14)	0.985 (0.774–1.254)	5 (0–11)	0.792 (0.581–1.083)	.024
Emergency OF fixation	1 (0–6)	1 (0–5)	0.630 (0.336–1.154)	1 (0–5)	0.666 (0.384–1.151)	1 (0–4)	0.476 (0.237–0.965)	.371

Pre-COVID (Jan. 2018—Jan. 2020), EP (Feb.—May 2020), Recovery (Jun.—Nov. 2020), Postrecovery (Dec. 2020—Dec. 2021). Bold indicates statistically significant associations.

Abbreviation: IRR: incidence rate ratio.

a From Kruskal–Wallis tests comparing distributions of weekly counts across the four time periods.

When stratified by pre-operative ASA score, there were significant increases in the volumes of ASA 3 (median 5 versus 3 and 4 in pre-COVID and EP, respectively) and ASA 4 (median 2 and 3 versus 1 and 1.5 in pre-COVID and EP, respectively) in the recovery and postrecovery periods (Supplementary Table S2 and Supplementary Figure S1). When stratified by post-op diagnosis, there were significant increases in the volumes of EL for bowel obstruction in the recovery and postrecovery periods (median 5 and 5.5 versus 3 during pre-COVID and EP, respectively), and increase in volumes for hepatobiliary conditions from EP onwards (median 3–3.5 versus 2 during pre-COVID) (Supplementary Table S3 and Supplementary Figure S2).

Outcomes were better from the EP period onwards. The adjusted median LOS was lower during EP at 6.7 days (*P* = .002), but returned to about 8 days in the recovery and postrecovery periods (Table 3). ICU admission and 30-day mortality decreased in the recovery and postrecovery periods compared to the pre-COVID period [odds ratio (OR) (95% CI): 0.376 (0.268–0.520) and 0.384 (0.298–0.492), respectively for ICU admission, 0.310 (0.177–0.516) and 0.396 (0.273–0.568), respectively for 30-day mortality] (Table 3).

**Table 3.** Clinical outcomes of patients by procedures and time period.

Characteristic	Pre-Covid	EP	Recovery	Postrecovery
EL, *n*	1899	283	557	1147
LOS (days), median (range)				
Raw	6 (0–332)	5 (1–155)	6 (1–234)	6 (0–280)
Fitted [*P*-value]	8.3 (0.4–20.8)	6.7 (0.4–17.6) [**.002**]	8.0 (0.9–19.7) [.038]	8.3 (0.7–20.3) [.003]
ICU admission, *n* (%)	311 (16.4)	31 (11.0)	65 (11.7)	156 (13.6)
OR (95% CI) [*P*-value]	Ref	0.505 (0.319–0.777) [0.003]	0.376 (0.268–0.520) [**6.84** **×** **10^–9^**]	0.384 (0.298–0.492) [**7.71** **x** **10^–14^**]
ICU LOS (days), median (range)				
Raw	3.6 (0.1–71.2)	5.6 (0.7–23.5)	2.7 (0.2–27.4)	4.6 (0.3–39.5)
Fitted [*P*-value]	3.6 (1.0–6.3)	5.4 (3.9–8.0) [.076]	3.1 (0.6–5.0) [.040]	4.6 (1.8–7.0) [.614]
30-Day unplanned readmission, *n* (%)	157 (8.3)	21 (7.4)	44 (7.9)	76 (6.6)
OR (95% CI) [*P*-value]	Ref	0.894 (0.540–1.411) [.646]	0.866 (0.602–1.221) [.423]	0.701 (0.521–0.934) [.017]
30-Day mortality, *n* (%)	117 (6.2)	10 (3.5)	19 (3.4)	55 (4.8)
OR (95% CI) [*P*-value]	Ref	0.474 (0.223–0.911) [.036]	0.310 (0.177–0.516) [**1.62** **×** **10^–5^**]	0.396 (0.273–0.568) [**6.95** **×** **10^–7^**]
Emergency CS, *n*	770	111	188	327
LOS (days), median (range)				
Raw	4 (1–36)	4 (1–15)	4 (1–41)	3 (2–70)
Fitted [*P*-value]	4 (4–4)	4 (4–4) [1.00]	4 (4–4) [1.00]	3 (3–3) [.026]
ICU admission, *n* (%)	4 (0.5)	0	1 (0.5)	2 (0.6)
OR (95% CI) [*P*-value]	Ref	5.59 × 10^–8^ [.995]	0.506 (0.024–3.833) [.562]	0.378 (0.039–2.611) [.347]
ICU LOS (days), median (range)^a^	2.2 (0.6–1158^b^)	–	0.3 (0.3–0.3)	2.3 (1.0–3.5)
30-day unplanned readmission, *n* (%)	15 (1.9)	0	2 (1.1)	8 (2.4)
OR (95% CI) [*P*-value]	Ref	1.578 × 10^–7^ [.988]	0.531 (0.081–1.988) [.413]	1.235 (0.444–3.196) [.672]
30-day mortality, *n* (%)	0	0	0	0
Emergency of fixation, *n*	170	20	39	70
LOS (days), median (range)				
Raw	3.5 (0–155)	3 (1–61)	4 (1–59)	4 (0–97)
Fitted [p-value]	4.9 (1.1–10.1)	2.7 (2.0–13.0) [0.848]	6.7 (2.9–13.1) [0.651]	3.6 (1.5–13.5) [0.558]
ICU admission, *n* (%)	5 (2.9)	1 (5.0)	0	0
OR (95% CI) [*P*-value]	Ref	1.513 (0.042–1.784) [.773]	5.245 × 10^–9^ [.998]	4.902 × 10^–9^ [.997]
ICU LOS (days), median (range)^a^	11.4 (11.4–14.5)	0.9 (0.9–0.9)	–	–
30-Day unplanned readmission, *n* (%)	4 (2.4)	1 (5.0)	1 (2.6)	3 (4.3)
OR (95% CI) [*P*-value]	Ref	6.546 (0.247–7.891) [0.164]	1.406 (0.067–1.152) [0.775]	2.589 (0.388–1.633) [0.302]
30-Day mortality, *n* (%)	2 (1.2)	0	0	0

Pre-COVID (Jan. 2018—Jan. 2020), EP (Feb.—May 2020), Recovery (Jun.—Nov. 2020), Postrecovery (Dec. 2020—Dec. 2021). Bold indicates statistically significant associations.

For LOS and ICU LOS, the fitted values refer to the predicted median values from the respective median regression models, and *P*-values are bootstrapped *P*-values for EP, recovery and postrecovery periods relative to pre-COVID.

a Median regression not performed due to small sample size

b Censored on 31 December 2021 (one patient still in ICU).

### Emergency CS

There were no differences in age or ethnicity across the time periods but the proportion of ASA 2 and 3 were increased from EP onwards (57.8–61.2% versus 23.4% in pre-COVID for ASA 2 and 5.9–8.6% versus 1.8% in pre-COVID for ASA 3) (Table 1).

There was also no significant change in weekly volumes of CS across the COVID time periods (Table 2). However, when stratified by ASA scores, there was an increase in ASA 2 (median 3–4 versus 2 in pre-COVID) and a corresponding decrease in ASA 1 (median 2 versus 5 in pre-COVID) slightly before the start of the EP period (Supplementary Figure S1).

There were no differences in clinical outcomes across the COVID time periods (Table 3).

### Emergency OF fixations

There were no differences in demographic characteristics but there was a trend towards a decrease in ASA 2 and a corresponding increase in ASA 1 during EP, which stabilized from the recovery period onwards (Table 1).

There were no significant differences in the weekly volume across the four COVID time periods or differences in clinical outcomes across the time periods (Tables 2 and 3).

## Discussion

### Statement of principal findings

This is the first study investigating the impact of ES postponement on the volumes and outcomes of three bellwether procedures in Singapore during the COVID-19 pandemic period. We found no significant effects of the EP policy on overall volumes of emergency CS and OF fixations but observed an increase in volumes of EL after the postponement.

### Interpretation within the context of the wider literature

Among the three bellwether procedures, the EP policy affected primarily EL. CS were not included in the policy and thus acted as a control. OF fixations by definition were emergency procedures and therefore not affected by the policy. It was thus expected that the volumes of CS and OF fixations did not change throughout the four time periods. In fact, there was a trend of decreased volume of OF fixations from EP onwards, likely because COVID-19 movement restrictions resulted in less population mobility and risk of traffic injuries [30]. This is consistent with the drop in trauma surgery by 21–67% in a systematic review [9].

The increase in volume of EL in the recovery and postrecovery periods, comprised mainly of patients with ASA score >3 with bowel obstruction or hepatobiliary conditions. Conditions such as small bowel obstruction secondary to adhesions, evolving malignant obstruction or biliary diseases may have possibly deteriorated during the postponement and presented as emergency cases. In a US study, 10 out of 27 patients with diverticulitis who had surgery postponed subsequently required urgent surgery [31]. Hernia and gallstones are typical indications where ES would have been postponed but may present with complications later, such as strangulation and biliary sepsis, respectively. In a German study, these were among the most commonly postponed procedures and while the volume of emergency cholecystectomies decreased during COVID-19, its associated mortality increased [32]. In our study, the clinical outcomes were interestingly better from EP onwards despite higher volumes after EP. While the EP policy helped to preserve ICU bed capacity, surgeons were rarely deployed to look after COVID-19 patients and so resources for postsurgical care were preserved.

The surprising finding is that the increase persisted even during the postrecovery period. However, we were unable to analyse individual cases to determine the reasons. There were brief periods of EP to varying degrees during the postrecovery periods in response to new waves of COVID-19 infections and this might have led to repeated cycles of clearing backlogs and therefore a higher overall volume than pre-COVID. The decision to postpone surgery might also be patient-driven due to fears of contracting COVID-19 in the hospital [19]. Surgeons in Italy reported delays in presentation partially due to patient choice and that urgent cases were generally more challenging to manage due to these delays [33]. It is also possible that patients in Singapore avoided presenting to the hospital beyond the initial pandemic period until their condition worsened, leading to a greater volume of EL cases observed.

One interesting observation was the rather abrupt increase in the proportion of patients with ASA 2 and a corresponding decrease in ASA 1 among the emergency CS cases even before the start of EP. In the early pandemic period, only public hospitals in Singapore were designated to receive pregnant women with COVID-19. One explanation could be that fears of contracting COVID-19 have driven healthy pregnant women to transfer to private hospitals, leaving those with more comorbidities and the more complicated cases in SGH. However, the overall volume remained constant, and the corresponding inflow cannot be explained by the handful of COVID-19 positive pregnant patients that might have transferred to SGH from other hospitals.

Outcomes did not worsen for all three procedures throughout COVID-19, which is reassuring given the increase in EL volumes in the recovery and postrecovery periods. COVID-19 infection in the peri-operative period can worsen outcomes for patients undergoing surgery, such as higher mortality, longer length of stay, and complications [34]. The Delta wave brought a spike of cases in Singapore between September and November 2021 [35]. However, by then, 70% of the population had already received the full regimen of COVID-19 vaccines [36]. This could explain why we did not see any detectable increase in adverse outcomes. Unfortunately, we did not have the vaccination and COVID-19 infection status of individual patients to verify this.

### Implications for policy, practice, and research

This study provided valuable insights to the effects of ES postponement, one of many policy decisions in the early pandemic period to preserve healthcare resources and reduce cross-infection. Based on the patterns in EL, which is most affected by the policy among the three procedures, the policy does not appear to have resulted in patient harm but might have increased the workload on healthcare staff. ES postponement is therefore likely to be a viable option in a crisis for health systems which are well-resourced and flexible to pivot operations to ensure their capacity is not overwhelmed. However, the outcomes of a wider range of procedures should be studied to provide more informed insights on types of procedures that can be safely postponed in future crises. Notwithstanding, other options for increasing bed count, such as converting spaces within the hospital to temporary wards [37], are preferable since ES postponement can impact patient’s function and emotions even if outcomes are not worse.

### Strengths and limitations

There are several limitations to this study. First, the data we extracted were designed to answer our research question but may not be sufficient to explain the reasons for certain trends we observed. Secondly, the three bellwether procedures are indicators of surgical capacity of a healthcare system. While we have shown that the surgical capacity in SGH is not affected by COVID-19, the exact clinical consequences may differ by type of surgery and medical condition, which are not represented by the three bellwether procedures. We are concurrently conducting similar studies on breast cancer and colorectal surgery to obtain a more holistic perspective. Thirdly, the classification of elective and emergency procedures may sometimes be inaccurate as elective patients may be listed as emergency for logistical or scheduling reasons. Fourthly, patient preferences and health-seeking behaviour might have changed in view of COVID-19 restrictions and the hospitals’ policy to defer elective surgeries. Therefore, some patients might have gone to other hospitals, potentially biasing our results. Lastly, the definition of the recovery and therefore the postrecovery periods were based on modelling estimates and may be inaccurate. However, this is difficult to define even post hoc.

### Conclusions

In conclusion, ES postponement in the early COVID-19 pandemic did not affect volumes of emergency CS and OF fixations but led to an increase in volume in EL after the postponement. Outcomes were not worse throughout the entire period. Nevertheless, it would be valuable for healthcare systems to invest in data capture systems to track relevant outcomes in real time should such policies be implemented in future crises again to understand the impact and therefore adjust strategies in a timelier fashion.

## Supplementary Material

mzae022_Supp

## Data Availability

The data underlying this article cannot be shared publicly due to privacy or ethical restrictions.
