# Supplementary material for: The impact of elective surgery postponement during COVID-19 on emergency bellwether procedures in a large tertiary centre in Singapore
Source: Int J Qual Health Care. 2024 Mar 20;36(1):mzae022. doi: 10.1093/intqhc/mzae022 (PMC10958764; doi:10.1093/intqhc/mzae022)
Supplement: mzae022_Supp [file mzae022_supp.zip › suppl_data/Table S1.docx]

Table S1 List of procedure codes for the 3 bellwether procedures

| Procedure | Procedure Code | Procedure description |
| --- | --- | --- |
| Laparotomy | SF700L | Liver transplant, donation (cadaveric donor) |
| Laparotomy | SF701C | Colon, anterior resection (open/mis) |
| Laparotomy | SF701G | Gallbladder, tumour, radical cholecystectomy with segment 4b and 5 liver resection and radical lymphadenectomy |
| Laparotomy | SF701L | Liver transplant, donation (live donor) |
| Laparotomy | SF701R | Rectum, resection - rectopexy for rectal prolapse (open or laparascopic) |
| Laparotomy | SF702G | Gallbladder, tumour, radical cholecystectomy with segment 4b and 5 liver resection and radical lymphadenectomy and choledochectomy with hepatico-enteric anastomosis |
| Laparotomy | SF702L | Liver transplant, recipient |
| Laparotomy | SF703G | Gallbladder, various lesions, cholecystectomy and repair of cholecysto-enteric/ cholecysto-choledochal fistula (open or lap) |
| Laparotomy | SF703R | Rectum, ultra-low anterior resection (total mesorectal excision) with/without plnd |
| Laparotomy | SF704A | Abdomen, laparotomy, trauma reoperation |
| Laparotomy | SF704G | Gallbladder, various lesions, cholecystectomy with intraoperative cholangiogram (open or lap) |
| Laparotomy | SF704P | Pancreas, various lesions, duodenum preserving pancreatic head resection with longitudinal pancreatico-enteric anastomosis (frey's operation) (sf704p) |
| Laparotomy | SF705A | Abdomen, laparotomy, trauma, damage control, haemostasis, packing |
| Laparotomy | SF705G | Gallbladder, various lesions, open/laparoscopic cholecystectomy and transcystic common bile duct exploration |
| Laparotomy | SF705P | Pancreas, various lesions, enucleation of lesions (1 to 2) |
| Laparotomy | SF706G | Gallbladder (acute/complicated) open or laparascopic cholecyestectomy |
| Laparotomy | SF706I | Intestine, small bowel, various lesions, extensive resection with anastomoses, with or without stoma (mis/open) |
| Laparotomy | SF706P | Pancreas, various lesions, enucleation of lesions (3 or more) (sf706p) |
| Laparotomy | SF706S | Stomach, various lesions, total/proximal gastrectomy with/without splenectomy |
| Laparotomy | SF707A | Abdomen, retroperitoneum, abscess, drainage with/without laparotomy |
| Laparotomy | SF707G | Gallbladder, various lesions, cholecystectomy, choledochotomy, common bile duct exploration with choledocho-duodenostomy (open or lap) |
| Laparotomy | SF707I | Intestine, small bowel, various lesions, simple resection with anastomoses, with or without stoma (mis/open) |
| Laparotomy | SF708G | Radical choledochectomy with liver resection (less than 4 segments) |
| Laparotomy | SF708I | Intestine, small bowel, vitello-intestinal fistula, excision |
| Laparotomy | SF708P | Pancreas, various lesions, mis/open distal pancreatectomy and splenectomy |
| Laparotomy | SF709A | Abdominal cavity, recurrent abdominal tumor, resection with complete abdominopelvic peritonectomy and hyperthermic intraoperative chemotherapy |
| Laparotomy | SF709G | Radical choledochectomy with liver resections (4 or more segments) or with multiple hepaticoenteric anastomosis |
| Laparotomy | SF709P | Pancreas, various lesions, mis/open subtotal pancreatectomy (extending to the neck) and splenectomy |
| Laparotomy | SF709S | Stomach, tumour, subtotal gastrectomy and radical lymphadenectomy |
| Laparotomy | SF710S | Stomach, tumour, total gastrectomy and radical lymphadenectomy |
| Laparotomy | SF711P | Pancreas, various lesions, segmental pancreatectomy with pancreatico-enteric anastomosis |
| Laparotomy | SF712C | Colon, total colectomy / subtotal colectomy |
| Laparotomy | SF712P | Pancreas, various lesions, mis/open spleen-preserving distal pancreatectomy |
| Laparotomy | SF712S | Stomach/duodenum, perforated ulcer, repair (mis or open) |
| Laparotomy | SF713C | Colon, total proctocolectomy & ileo-anal pouch reconstruction |
| Laparotomy | SF723A | Appendix, various lesions/abscess, appendicectomy with drainage (mis/open) |
| Laparotomy | SF800A | Abdominal cavity, adhesions (extensive), lysis as primary procedure, extensive with or without bowel resection (laparoscopic or open) where time taken is more than 2hours |
| Laparotomy | SF800C | Colon, colostomy, closure without resection of bowel |
| Laparotomy | SF800D | Diaphragm, diaphragmatic/hiatus hernia, repair (mis/open) |
| Laparotomy | SF800I | Intestine, enterostomy, closure |
| Laparotomy | SF801A | Abdominal cavity, adhesions (limited), lysis as primary procedure (mis/open) where time taken is less than 2hours |
| Laparotomy | SF801B | Bile duct, various lesions, choledocho-duodenostomy/choledocho-gastrostomy |
| Laparotomy | SF801D | Duodenum various lesions, gastroenterostomy/gastroduodenostomy |
| Laparotomy | SF801G | Gall bladder, various lesions, cholecystectomy (open or lap) |
| Laparotomy | SF802A | Abdominal cavity, lymphoma,for grading lymphoma with splenectomy/liver/lymph node biopsy (mis/open) |
| Laparotomy | SF802B | Bile duct, various lesions, choledocho-jejunostomy |
| Laparotomy | SF802C | Colon, various lesions, colostomy |
| Laparotomy | SF802D | Duodenum, trauma, bypass operation (sf802d) |
| Laparotomy | SF802G | Gall bladder, various lesions, cholecystectomy and choledocho-jejunostomy/duodenostomy/gastrostomy |
| Laparotomy | SF803A | Abdominal cavity, multiple ruptures, major repair/removal |
| Laparotomy | SF803B | Bile duct, various lesions, choledochotomy (exploration common bile duct only) |
| Laparotomy | SF803C | Colon, various lesions, right/left hemicolectomy (mis/open) |
| Laparotomy | SF803G | Gall bladder, various lesions, cholecysto-duodenostomy/cholecysto-gastrectomy/cholecysto jejunostomy |
| Laparotomy | SF803S | Stomach, morbid obesity, bypass only |
| Laparotomy | SF804B | Bile duct, various lesions. Hepaticojejunostomy up to but not including the confluence of the hepatic ducts |
| Laparotomy | SF804C | Colon, various lesions, total colectomy with ileorectal anastomosis/ileostomy |
| Laparotomy | SF804E | Esophagus, tumor, allison/ivor-lewis operation |
| Laparotomy | SF804L | Liver, abscess, trans-abdominal drainage (open or lap) |
| Laparotomy | SF804S | Stomach, morbid obesity, reduction and bypass (mis/open) |
| Laparotomy | SF805C | Colon, various lesions, total procto-colectomy and ileostomy |
| Laparotomy | SF805E | Esophagus, tumor, bypass with stomach/intestine (mis/open) |
| Laparotomy | SF805I | Intestine, meckel's diverticulum, various lesions, resection (lap/open) |
| Laparotomy | SF805P | Pancreas, cyst/pseudocyst, mis/open drainage and anastomosis to intestine/stomach (sf805p) |
| Laparotomy | SF805R | Rectum, tumor, anterior resection/abdomino -perineal resection with salpingo-oophorectomy and total hysterectomy |
| Laparotomy | SF806A | Abdominal cavity, ruptured viscus, simple repair |
| Laparotomy | SF806B | Bile duct, various lesions, revision high biliary stricture/ radical resection |
| Laparotomy | SF806C | Colon, various lesions, tranverse/sigmoid colectomy (mis/open) |
| Laparotomy | SF806P | Pancreas, tumour, triple bypass |
| Laparotomy | SF807A | Abdominal cavity, subphrenic abscess, drainage |
| Laparotomy | SF807P | Pancreas, various lesions, distal pancreatectomy/anastomosis of pancreatic duct |
| Laparotomy | SF807R | Rectum, various lesions, abdomino-perineal pull through resection with colo-anal anastomosis |
| Laparotomy | SF808A | Abdominal cavity, various lesions, exploratory laparotomy (mis/open) |
| Laparotomy | SF808B | Bile duct, various lesions, hepaticojejunostomy involving anastomosis to hepatic ducts but without liver resection |
| Laparotomy | SF808R | Rectum, various lesions, hartmann's procedure |
| Laparotomy | SF808S | Stomach, tumor/ulcer, gastrojejunostomy |
| Laparotomy | SF809A | Abdominal cavity, various lesions, including colostomy/enterostomy/gastrostomy (mis/open) |
| Laparotomy | SF809E | Esophagus, tumor, total esophagectomy |
| Laparotomy | SF809L | Liver, trauma/tumor, extended lobectomy (5 segments/more) |
| Laparotomy | SF809P | Pancreas, various lesions, whipple operation/total pancreatectomy |
| Laparotomy | SF809S | Stomach, ulcers, vagotomy - all types (mis/open) |
| Laparotomy | SF810A | Abdominal cavity, various lesions, not classified elsewhere (mis/open) |
| Laparotomy | SF810S | Stomach, various lesions, partial/subtotal gastrectomy |
| Laparotomy | SF811S | Stomach, various lesions/post-gastrectomy complications, revision gastrectomy (sf811s) |
| Laparotomy | SF812E | Esophagus, various lesions, cervical esophagostomy (sf812e) |
| Laparotomy | SF812L | Liver, various lesions, lobectomy (3 - 4 segments), open or lap |
| Laparotomy | SF812S | Stomach, total/proximal gastrectomy for benign gastric diseases |
| Laparotomy | SF813A | Abdominal wall, burst abdomen, repair |
| Laparotomy | SF813L | Liver, various lesions, partial lobectomy/segmental resection (open or lap) |
| Laparotomy | SF813S | Stomach, various lesions, wedge resection |
| Laparotomy | SF814A | Abdominal wall, epigastric/umbilical hernia, repair (mis/open) |
| Laparotomy | SF815L | Liver, various lesions, wedge/local excision (open or lap) |
| Laparotomy | SF819A | Abdominal wall, inguinal/femoral hernia, unilateral herniorrhaphy (mis/open) |
| Laparotomy | SF820A | Abdominal wall, inguinal/femoral hernia, bilateral herniorrhaphy (mis/open) |
| Laparotomy | SF821A | Abdominal wall, strangulated/obstructed hernia, repair with bowel resection |
| Laparotomy | SF822A | Abdominal wall, strangulated/obstructed hernia, repair without bowel resection |
| Laparotomy | SF823A | Abdominal wall, ventral/incisional/recurrent hernia, repair (mis/open) |
| Laparotomy | SF832A | Anus, fistula-in-ano (high), complex and recurrent fistulectomy |
| Laparotomy | SF845A | Anus, tumor, abdomino-perineal resection (open or lap) |
| Laparotomy | SF848A | Appendix, tumor, right hemicolectomy with/without adhesiolysis (open or lap) |
| Laparotomy | SF849A | Appendix, various lesions, appendicectomy without drainage, mis/open |
| Caesarean | SI832U | Uterus, pregnancy and multiparity, uncomplicated caesarean section and tubal ligation |
| Caesarean | SI834U | Uterus, pregnancy, uncomplicated caesarean section |
| Caesarean | SI835U | Uterus, pregnancy, caesarean section with hysterectomy |
| Caesarean | SP832U | Uterus, pregnancy and multiparity, lower segment/classical caesarean section and tubal ligation |
| Caesarean | SP834U | Uterus, pregnancy, uncomplicated caesarean section |
| Caesarean | SP835U | Uterus, pregnancy, caesarean section with hysterectomy |
| Open Fracture | SB700A | Ankle, ankle fracture, bimalleolar, orif |
| Open Fracture | SB700R | Radius and ulna, fracture/dislocation, non union/delayed union bone grafting and re-fixation |
| Open Fracture | SB700U | Upper limb/lower limb, fracture, external fixation, multiple planes |
| Open Fracture | SB701A | Ankle, comminuted plafond fracture orif |
| Open Fracture | SB701C | Clavicle, clavicle fracture, comminuted plating with or without bone grafting |
| Open Fracture | SB701P | Pelvis, fracture, fixation of anterior and posterior ring |
| Open Fracture | SB701T | Tibia, fracture, open reduction and internal fixation with plates and screws, comminuted |
| Open Fracture | SB702E | Elbow, coronoid fracture, orif |
| Open Fracture | SB703F | Femur, fracture/orif with plate/screws, complex intra-articular fractures |
| Open Fracture | SB703H | Hand, closed fracture, orif complex (multiple) |
| Open Fracture | SB704H | Hand, closed fracture, orif/plate and screws (single), joint/non-joint |
| Open Fracture | SB705E | Elbow, fracture/dislocation, open reduction and internal fixation (orif) |
| Open Fracture | SB705F | Femur, fracture, multifragmentary, im nailing (with or without locking) |
| Open Fracture | SB705H | Hand, closed fracture, orif with k-wire/cerclage wire fixation |
| Open Fracture | SB705T | Tibia, tibial shaft fracture, im nailing (locked nail) |
| Open Fracture | SB705W | Wrist, distal radius fracture, orif - simple open reduction and pinning |
| Open Fracture | SB706H | Hand, complex injuries, debridement with repair/reconstruction (more than 2 tendons involved or more than 2 fractures) |
| Open Fracture | SB706W | Wrist, distal radius fracture, open reduction and internal fixation (orif) (complex, with autologous bone graft) |
| Open Fracture | SB707W | Wrist, distal radius fracture, open reduction and internal fixation (orif) (with ulnar head/styloid fixation/tfcc repair) |
| Open Fracture | SB709F | Femur, single condylar fracture, orif |
| Open Fracture | SB710H | Hand, fracture of fingers/metacarpals, external fixation |
| Open Fracture | SB710W | Wrist, scaphoid fracture, open reduction and internal fixation (orif) with bone grafting |
| Open Fracture | SB711H | Hand, fracture of fingers/metacarpals, non-union, bone-grafting and fixation (multiple) |
| Open Fracture | SB712H | Hand, fracture of fingers/metacarpals, non-union, bone-grafting and fixation (single) |
| Open Fracture | SB713H | Hand, closed fracture, orif (multiple), joint |
| Open Fracture | SB714F | Forearm, radius/ulna shaft fracture, intramedullary rod |
| Open Fracture | SB714H | Hand, open fracture, debridement and orif (multiple), joint |
| Open Fracture | SB715H | Hand, open fracture, debridement and orif (multiple), non-joint |
| Open Fracture | SB716H | Hand, open fracture, debridement and orif (single), joint |
| Open Fracture | SB717H | Hand, open fracture, debridement and orif (single), non-joint |
| Open Fracture | SB718J | Joints, major intra-articular fracture with discontinuity or bone loss, reconstruction (for upper limb) |
| Open Fracture | SB721F | Foot, fractures, complex, multiple, including talus and calcaneum, open reduction and internal fixation |
| Open Fracture | SB722F | Foot, fractures, simple, single |
| Open Fracture | SB730H | Humerus, humerus shaft fracture, complex multi-fragmentary intramedullary nail |
| Open Fracture | SB800P | Patella, fracture, open reduction and internal fixation |
| Open Fracture | SB801A | Ankle, ankle fracture, unimalleolar, orif |
| Open Fracture | SB801C | Carpus, fracture/dislocation, open reduction and internal fixation |
| Open Fracture | SB801E | Elbow, fracture/ dislocation, repair of ligaments/ orif |
| Open Fracture | SB801H | Hand, closed fracture/dislocation, open reduction and fixation (single) |
| Open Fracture | SB801R | Radius and ulna, fracture/dislocation, open reduction and internal fixation with or without bone grafting |
| Open Fracture | SB802H | Hand, complex injuries, debridement with repair/reconstruction (1-2 tendons involved or 1-2 fractures) |
| Open Fracture | SB804B | Bone (lower limb), fracture/dislocation, manipulation and kirschner wire fixation |
| Open Fracture | SB805B | Bone (lower limb), grade 1 and 2 open fractures/open dislocation or open fracture-dislocation wound debridement |
| Open Fracture | SB806M | Mandible, fracture, open reduction and fixation |
| Open Fracture | SB807L | Upper limb/lower limb, open fractures with debridement and external fixation |
| Open Fracture | SB810F | Femur, fracture (femur shaft), intramedullary nailing/rod (with or without locking) |
| Open Fracture | SB811F | Femur, fracture, open reduction and internal fixation with plate and screws |
| Open Fracture | SB814H | Hand, fractures/dislocation, open reduction and internal fixation (multiple) |
| Open Fracture | SB822B | Bone (upper limb), grade 1 and 2 open fractures/open dislocation or open fracture-dislocation wound debridement |
| Open Fracture | SB827T | Tibia, fracture(s), open reduction and internal fixation with intramedullary rod |
| Open Fracture | SB828T | Tibia, fracture, open reduction and internal fixation with plates and screws, simple |
| Open Fracture | SB828T | Tibia, fracture, open reduction and internal fixation with plates and screws, simple |
| Open Fracture | SB842H | Humerus supracondylar and intercondylar fracture |
| Open Fracture | SC706T | Thorax, ribs, open fixation of rib fractures (>3 ribs) |
