# Supplementary material for: The impact of elective surgery postponement during COVID-19 on emergency bellwether procedures in a large tertiary centre in Singapore
Source: Int J Qual Health Care. 2024 Mar 20;36(1):mzae022. doi: 10.1093/intqhc/mzae022 (PMC10958764; doi:10.1093/intqhc/mzae022)
Supplement: mzae022_Supp [file mzae022_supp.zip › suppl_data/Table S2 rev.docx]

Table S2 Weekly volumes by type of procedure, priority and ASA Status

| Surgery | ASA Status | Median (range) | | | | P value |
| --- | --- | --- | --- | --- | --- | --- |
|  |  | Pre-COVID | EP | Recovery | Post-recovery |  |
| EL | 1 | 4  (1 – 8) | 4  (1 – 8) | 3.5  (2 – 10) | 3  (1 – 7) | 0.798 |
|  | 2 | 8  (3 – 19) | 7  (4 – 13) | 9  (5 – 22) | 9  (3 – 15) | 0.182 |
|  | 3 | 3  (1 – 13) | 4  (1 – 8) | 5  (2 – 11) | 5  (1 – 14) | **1.18 x 10^-5^** |
|  | 4 | 1  (1 – 5) | 1.5  (1 – 3) | 2  (1 – 6) | 3  (1 – 7) | **1.36 x 10^-6^** |
|  | 5 | 1 (1 – 1) | 1 (1 – 1) | 1 (1 – 1) | 1 (1 – 1) |  |
| Emergency CS | 1 | 5 (1 – 12) | 2 (0 – 6) | 2 (1 – 5) | 2 (0 – 4) | **<2.2 x 10^-16^** |
|  | 2 | 2  (1 – 8) | 4  (1 – 7) | 4  (1 – 11) | 3  (1 – 7) | **3.06 x 10^-8^** |
|  | 3 | 1 (1 – 1) | 1 (1 – 4) | 1 (1 - 2) | 1 (1 – 3) | 0.200 |
|  | 4 | 1 (1 – 1) | - | 1.5 (1 – 2) | 1 (1 – 2) | 0.333 |
| Emergency OF fixation | 1 | 1 (0 – 5) | 1 (1 – 4) | 1 (0 – 4) | 1 (0 – 2) | 0.806 |
|  | 2 | 1 (1 – 4) | 1 (1 – 1) | 2 (1 – 4) | 1 (1 – 2) | 0.070 |
|  | 3 | 1 (1 – 1) | 1 (1 – 1) | 1 (1 – 1) | 1 (1 – 2) | 0.615 |
|  | 4 | 0 | 1 (1 – 1) | 1 (1 – 1) | 1 (1 – 1) |  |

Pre-COVID (Jan 2018 – Jan 2020), EP (Feb – May 2020), Recovery (Jun – Nov 2020), Post-recovery (Dec 2020 – Dec 2021)

ASA: American Society of Anesthesiologists, CS: Caesarean section, EL: emergency laparotomy, EP: elective postponement, OF: open fracture
