# Supplementary material for: The impact of elective surgery postponement during COVID-19 on emergency bellwether procedures in a large tertiary centre in Singapore
Source: Int J Qual Health Care. 2024 Mar 20;36(1):mzae022. doi: 10.1093/intqhc/mzae022 (PMC10958764; doi:10.1093/intqhc/mzae022)
Supplement: mzae022_Supp [file mzae022_supp.zip › suppl_data/Table S3 rev.docx]

Table S3 Weekly volume of laparotomies by post-operative diagnosis

| Diagnosis | Median (range) | | | | P value |
| --- | --- | --- | --- | --- | --- |
|  | Pre-COVID | EP | Recovery | Post-recovery |  |
| Appendicitis | 6  (1 – 14) | 7  (3 – 11) | 7  (2 – 13) | 7  (3 – 16) | 0.290 |
| Bowel obstruction | 3  (1 – 9) | 3  (2 – 6) | 5  (1 – 9) | 5.5  (1 -9) | **1.38 x 10^-5^** |
| Hepatobiliary conditions | 2  (1 – 8) | 3.5  (1 – 7) | 3  (1 – 17) | 3  (1 – 7) | **9.03 x 10^-5^** |
| Hernia | 1 (1 – 4) | 1 (1 – 2) | 2 (1 – 4) | 2 (1 – 4) | 0.252 |
| Ischemic bowel | 1 (1 – 3) | 1 (1 – 3) | 1 (1 – 3) | 1 (1 – 4) | 0.901 |
| Perforation | 2  (1 – 6) | 2  (1 – 5) | 2  (1 – 5) | 2  (1 – 6) | 0.685 |

Pre-COVID (Jan 2018 – Jan 2020), EP (Feb – May 2020), Recovery (Jun – Nov 2020), Post-recovery (Dec 2020 – Dec 2021)

EP: elective postponement
