# Supplementary figures and images for: The impact of elective surgery postponement during COVID-19 on emergency bellwether procedures in a large tertiary centre in Singapore
Source: Int J Qual Health Care. 2024 Mar 20;36(1):mzae022. doi: 10.1093/intqhc/mzae022 (PMC10958764; doi:10.1093/intqhc/mzae022)

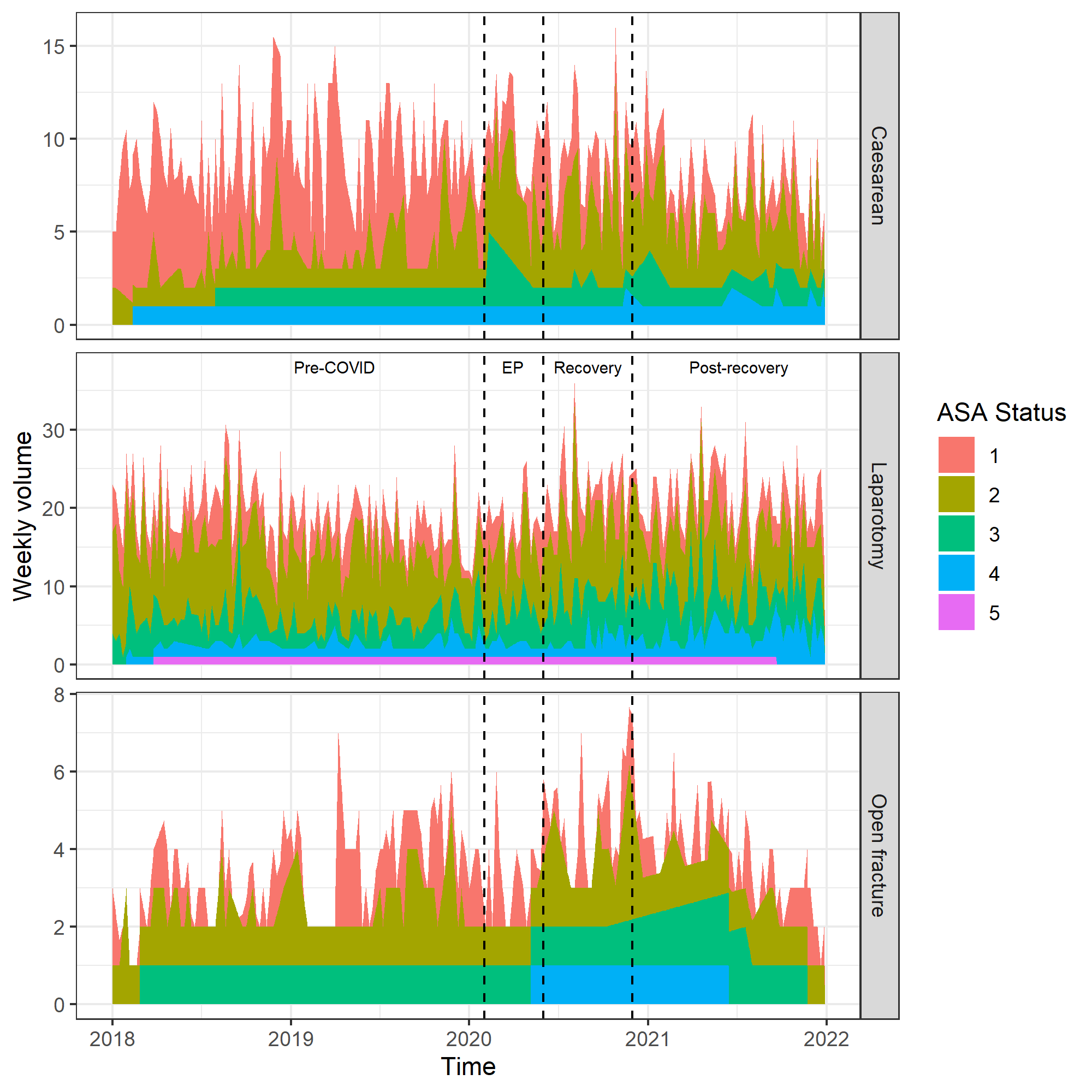

Supplement: mzae022_Supp [file mzae022_supp.zip › suppl_data/Supp_Fig_1_rev.png]

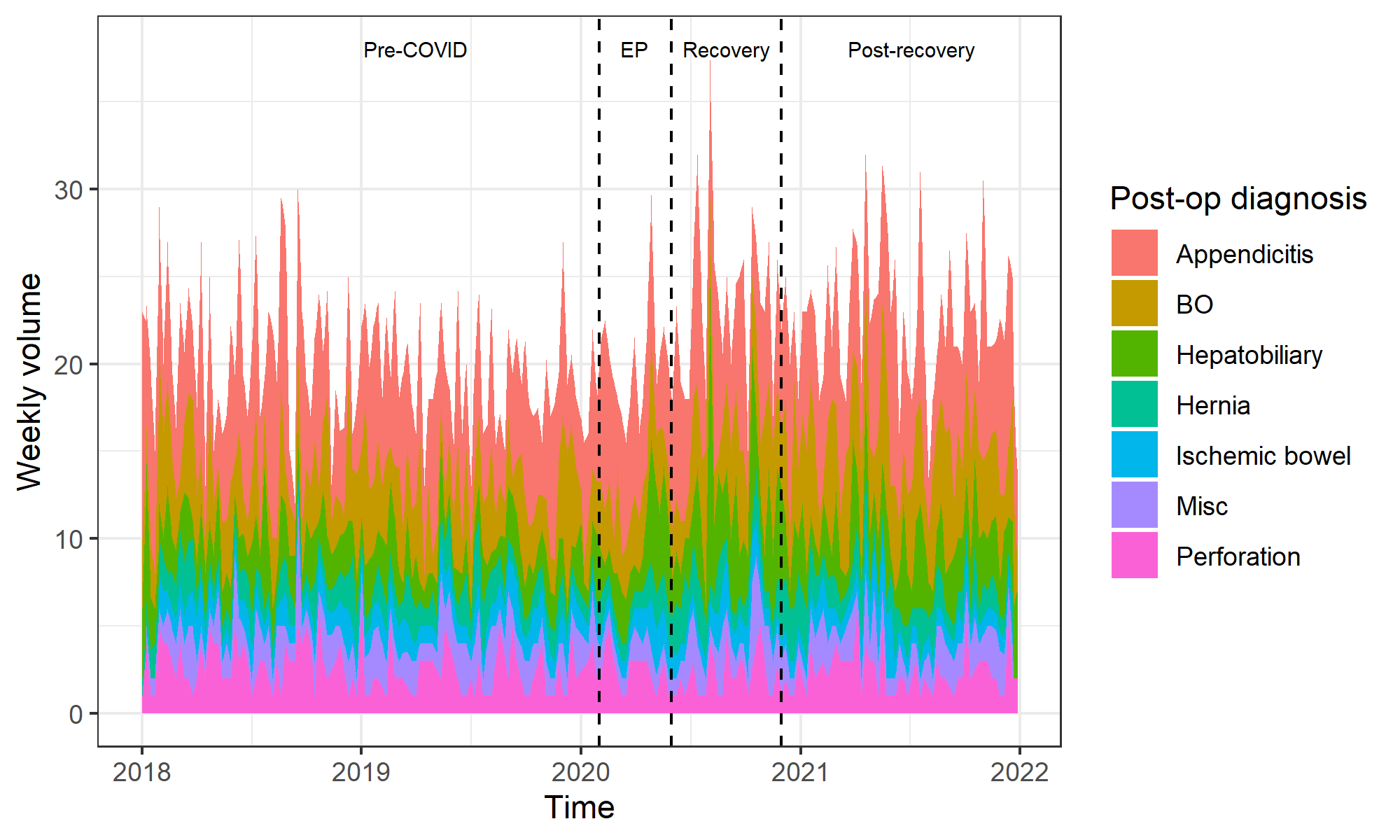

Supplement: mzae022_Supp [file mzae022_supp.zip › suppl_data/Supp_fig_2_rev.png]
